# Diagnostic Accuracy of Artificial Intelligence Software in Intraoral, Panoramic, and CBCT Imaging: A Pilot Study

**DOI:** 10.3390/jcm15207835

**Published:** 2026-10-11

**Authors:** Wojciech Czyż, Ingrid Różyło-Kalinowska, Paweł Dolibog, Małgorzata Skucha-Nowak

**Affiliations:** 1 Faculty of Medical Sciences in Zabrze, Medical University of Silesia in Katowice, 40-055 Katowice, Poland; 2 Department of Dental and Maxillofacial Radiodiagnostics, Faculty of Medical Dentistry, Medical University of Lublin, 20-093 Lublin, Poland; ingrid.rozylo-kalinowska@umlub.edu.pl; 3 Department of Biophysics, Faculty of Medical Sciences in Zabrze, Medical University of Silesia in Katowice, 40-055 Katowice, Poland; pawel.dolibog@sum.edu.pl; 4 Department of Dental Propedeutics, Faculty of Medical Sciences in Zabrze, Medical University of Silesia in Katowice, 40-055 Katowice, Poland; mskucha-nowak@sum.edu.pl

**Keywords:** artificial intelligence, dental radiology, intraoral radiographs, panoramic radiograph, cone-beam computed tomography, diagnostic imaging, diagnostic accuracy

## Abstract

**Background/Objectives:** Artificial intelligence (AI) is increasingly used in dental radiology, but its performance may depend on the imaging modality and the analytical model. This pilot (feasibility) study evaluated the diagnostic accuracy of AI-based software (Diagnocat) in intraoral, panoramic, and cone-beam computed tomography (CBCT) images against a single-expert reference standard, accounting both for indications of pathology and for indications of its absence. **Methods:** In this retrospective analysis, 45 anonymized examinations (15 intraoral radiographs, 15 panoramic radiographs, and 15 CBCT) were assessed. The reference standard was established by an expert in dental and maxillofacial radiology with over 30 years of experience, who took no part in selecting the examinations and was blinded to the software output. The primary analysis was performed per diagnostic indication, defined as the combination of one examination, one diagnostic category, and one anatomical location. A secondary binary “pathology versus no pathology” analysis was performed with one observation per tooth or anatomical site. Because observations were clustered within examinations and patients, the analysis was descriptive; no hypothesis tests were performed, and no confidence intervals were reported. **Results:** In the primary per-indication analysis, 999 diagnostic indications were recorded in the reference standard and 1017 by the software; 791 were concordant, comprising 45 of 71 reference indications for intraoral radiographs, 358 of 452 for panoramic radiographs, and 388 of 476 for CBCT. In the secondary binary analysis, intraoral radiographs yielded sensitivity 0.79, specificity 0.59, precision 0.71, and F1-score (the harmonic mean of sensitivity and precision) 0.75; panoramic radiographs 0.73, 0.88, 0.79, and 0.75; and CBCT 0.80, 0.86, 0.78, and 0.79. Sensitivity and F1-score were numerically highest for CBCT. The three modality groups comprised different patients, anatomical regions, and distributions of diagnostic categories, so these differences cannot be attributed to the imaging modality alone. **Conclusions:** In this pilot study, the performance of the software varied across imaging modalities and diagnostic categories. Because the set of negative units was defined by the locations charted by the expert rather than by a complete anatomical inventory, specificity, negative predictive value (NPV), and accuracy are reported as secondary descriptive outcomes, are conditional on that operational definition, and are not compared between modalities. The findings are exploratory and hypothesis-generating. The software may support the interpretation of dental radiographs, but should not replace expert assessment.

## 1. Introduction

Artificial intelligence (AI) is finding increasingly wide application in medicine, including diagnostic imaging of the oral cavity and maxillofacial structures. In dental radiology, systems are being developed for the detection and classification of pathology, the segmentation of anatomical structures, the improvement of image quality, and the support of treatment planning. Published studies indicate that these applications cover an increasingly broad spectrum of diagnostic tasks, but that their clinical usefulness still requires critical evaluation [1,2,3].

Dental radiology is based on several fundamental imaging modalities that differ in the range of visible structures and the amount of diagnostic information provided. Intraoral radiographs allow a detailed assessment of individual teeth and periapical tissues; panoramic radiographs enable a general assessment of the dentition and some maxillofacial structures in a single image; and cone-beam computed tomography (CBCT) provides three-dimensional data of the highest degree of complexity. It can therefore be assumed that the performance of AI systems will depend not only on the type of analyzed pathology, but also on the type and area of imaging [1,4]. Diagnostic performance also depends on the physical characteristics of the target rather than on the imaging modality alone, so the three modalities may play complementary roles.

The results of studies on AI algorithms in dental radiology published to date have most often focused on single image types and selected diagnostic tasks. For intraoral radiographs, the detection of periapical lesions, periodontal bone loss, and endodontic complications, among others, has been analyzed. In panoramic imaging, the recognition of dental pathologies, developmental changes, and abnormalities visible across the whole dentition has been assessed. In CBCT, particular attention has been paid to periapical lesions and the assessment of dental structures, as well as to more complex analytical tasks such as the segmentation of anatomical structures associated with three-dimensional imaging [4,5,6,7,8].

Diagnocat (DGNCT LLC, Miami, FL, USA) is a cloud-based, convolutional-neural-network system marketed for the automated analysis of intraoral, panoramic, and CBCT images. In dentistry, it is used to flag common radiological findings, such as caries, periapical lesions, periodontal bone loss, and missing teeth, and to generate structured radiographic reports. It has been validated in several single-modality studies, most often for panoramic radiographs or CBCT [9,10,11], with performance that varied markedly by diagnostic task; however, these evaluations have generally been confined to one imaging type at a time.

Despite the growing number of publications, an important limitation of the literature to date remains its fragmentary nature. Most studies concern a single imaging type, a single category of pathology, or a single predefined diagnostic task. This makes a direct comparison of AI performance across different clinical conditions difficult. In addition, in many studies, the unit of analysis is limited exclusively to pathological changes, without considering situations in which the correct result is the statement of the absence of pathology. Yet in clinical practice, the correct recognition of a case without pathology is just as important as the detection of a lesion [2,4,6]. Without correctly recognized cases of absent pathology, there is no true-negative count, and specificity cannot be estimated; yet in daily practice, the confident exclusion of pathology is as consequential as its detection, since it underlies decisions not to treat and helps to avoid overdiagnosis.

From the point of view of the clinical usefulness of AI-based software, it is therefore important to determine not only whether the system can detect pathology, but also whether it remains consistent with the expert assessment across different imaging modalities and whether it correctly marks the absence of pathology where it is genuinely not present. This kind of approach allows a more accurate definition of the diagnostic profile of the tool under study and better reflects the conditions of everyday dental practice [4,5,6]. To our knowledge, no study has evaluated a single AI system across intraoral, panoramic, and CBCT imaging within one analytical framework that also records the correct identification of absent pathology, and this is the gap the present pilot study addresses. The claim should be read with one qualification: the true-negative component of such a framework depends on how the set of negative units is defined, and in this study, it followed from the locations charted by the expert rather than from a predefined anatomical inventory. The measures derived from it are therefore reported as secondary and conditional.

The aim of the present study was to evaluate the diagnostic accuracy of the AI-based software Diagnocat in the analysis of dental radiological images across three imaging modalities—intraoral radiographs, panoramic radiographs, and CBCT—compared with expert assessment, accounting for both the presence of pathological changes and the “no pathology” indication. An additional aim was to describe the results separately for each imaging type. Because the three groups comprised different patients and different anatomical regions, no formal comparison between modalities was planned as an endpoint. We hypothesized that diagnostic accuracy would vary across modalities and diagnostic categories, with the lowest performance expected for subtle two-dimensional findings; the primary outcome was per-indication agreement between the AI software and the expert reference standard. The study is reported in accordance with the STARD 2015 guidelines (Supplementary Material S1).

## 2. Materials and Methods

### 2.1. Study Design and Material

**Figure 1:**
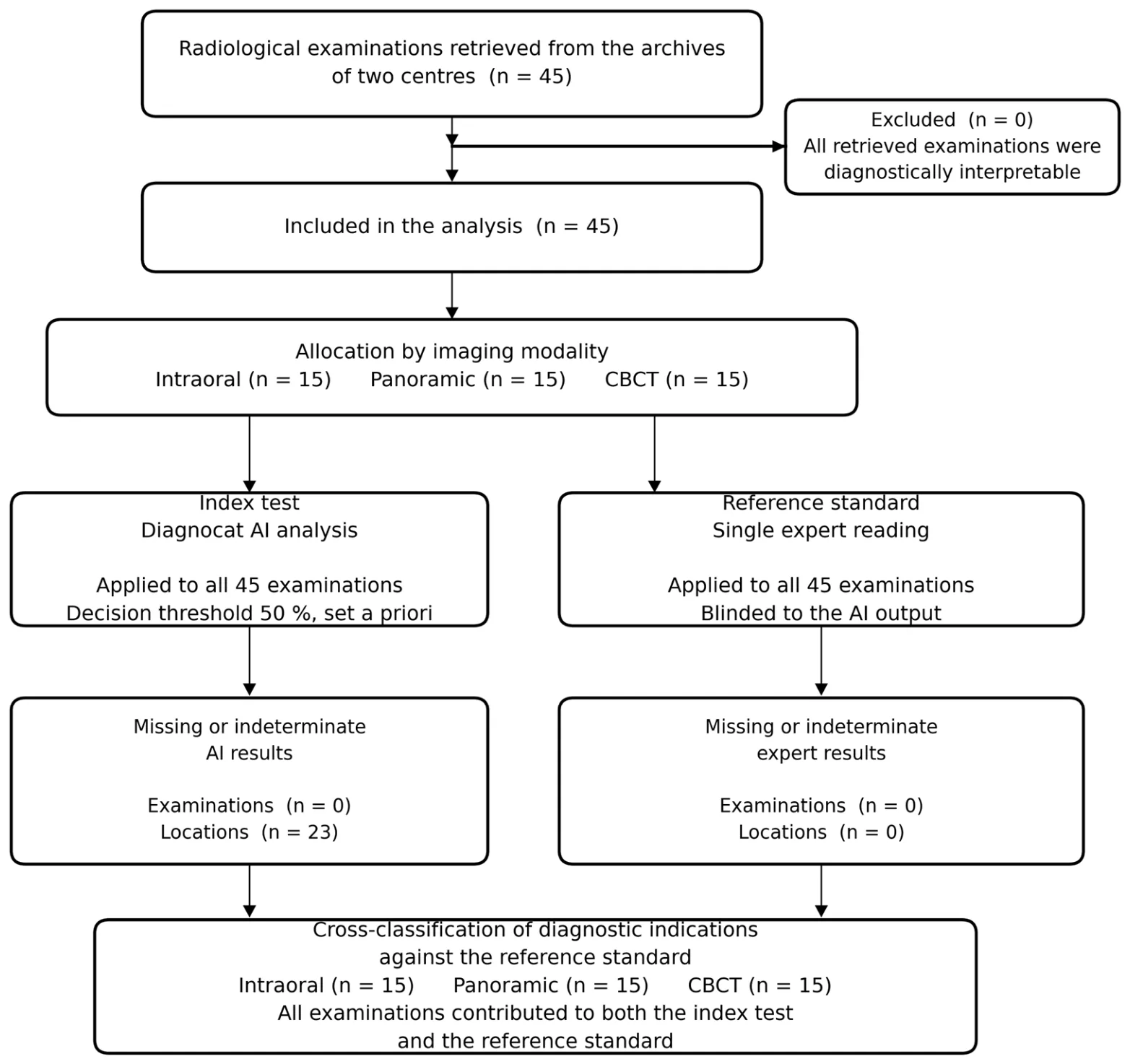
Study flow diagram prepared in accordance with the STARD 2015 recommendations. All 45 retrieved examinations were eligible and were included; no examinations were excluded. Both the index test (Diagnocat) and the reference standard (expert reading) were applied to every examination, and no examination produced an indeterminate result. At the level of the individual location, the software produced no entry at 23 of the locations charted by the expert; these are treated as missing index test results and are listed in Supplementary Material S2.

The study was retrospective and performed on anonymized radiological material from two centers: the Academic Center of Dentistry (Bytom, Poland) and the Skrzelewska-Czyż Center of Dentistry and Medicine (Goczałkowice-Zdrój, Poland). This study was designed as a pilot (feasibility) study. The sample of 15 examinations per modality (45 in total) was chosen deliberately as a feasibility sample intended to test the analytical framework and to provide preliminary estimates of diagnostic accuracy; no formal sample-size (power) calculation was performed, and the results should be interpreted as preliminary and hypothesis-generating rather than confirmatory. A total of 45 imaging examinations were included—15 intraoral radiographs, 15 panoramic radiographs, and 15 cone-beam computed tomography (CBCT) examinations. Examinations were selected for inclusion by one investigator (W.C.) in a non-systematic (convenience) manner, without a formal randomization procedure, and independently of the presence or absence of pathology; only anonymized, diagnostic-quality examinations were considered, and indications related to tooth germs were not assessed. The analysis in the Diagnocat system was carried out in December 2025. The study received a favorable opinion from the Bioethics Committee of the Medical University of Silesia in Katowice (approval number BNW/NWN/0052/KB/316/25; date of approval 20 October 2025). Each examination originated from a separate patient (45 patients in total). Because the material was fully anonymized before analysis, patient age and sex were not available. Examinations were drawn from both participating centers across all three modalities and were selected by convenience rather than consecutively or at random, so that selection bias cannot be excluded; this further supports interpreting the findings as exploratory. An examination was considered of diagnostic quality when the region of interest was fully captured and free of motion, positioning, or exposure artifacts that would preclude confident interpretation, and all 45 selected examinations met this standard. In total, 45 examinations were retrieved, and none were excluded; all 45 (15 intraoral, 15 panoramic, and 15 CBCT) were included in the analysis (Figure 1). The study is reported in accordance with the STARD 2015 recommendations; the completed checklist is provided as Supplementary Material S1.

Intraoral and panoramic radiographs were analyzed in JPEG format, whereas CBCT examinations were analyzed in DICOM format. All images were anonymized before the analysis began. Neither the AI software nor the expert creating the reference standard had access to the patients’ clinical data. Images were acquired on Dentsply Sirona equipment (Bensheim, Germany); because the material was retrospective and anonymized, detailed acquisition parameters (e.g., field of view, voxel size, and exposure settings) were not retrievable, which limits full reproducibility. No restriction was placed on the type of pathology present, and examinations containing developmental abnormalities were eligible. The decision threshold applied to the software output was set at 50% a priori by the investigators; it is not a manufacturer-defined default operating point, and no threshold optimization or ROC analysis was performed. The center of origin was not recorded in the anonymized dataset, and the numerical distribution of examinations between centers could not be reconstructed; likewise, the acquisition parameters of the individual examinations, including device models, the intraoral image receptor, the panoramic acquisition settings, and the CBCT field of view and voxel size, could not be retrieved from the archive. Both omissions are stated among the limitations.

### 2.2. Reference Standard

The reference standard was established on the basis of the assessment of whole images by an expert in dental and maxillofacial radiology and diagnostic imaging with more than 30 years of clinical experience (I.R.-K.), who did not take part in selecting the examinations and who was blinded to the software output. The expert assessment comprised the identification and classification of all diagnostic indications present, as well as the marking of cases in which no pathology was found. The expert was blinded to the AI output and to the source center and patient identity and assessed each fully anonymized image in isolation.

### 2.3. AI Software

The Diagnocat software (DGNCT LLC, Miami, FL, USA), in the December 2025 version, was used for the analysis. Image analysis was performed automatically, in a uniform manner for all cases, and without intervention by the investigators, and the software was run in its factory configuration, with no parameters changed. The software reports a probability for each detected condition and does not itself classify it as present or absent, so a classification rule had to be defined by the investigators. The manufacturer was contacted and did not specify a recommended classification threshold. In the absence of such a recommendation, a cutoff of more than 50% was adopted a priori, corresponding to the “more likely than not” criterion, that is, to a situation in which the probability that the condition is present exceeds the probability of its absence. The 50% cutoff is therefore a study-specific decision rule and not a manufacturer-defined operating point. The same threshold was applied to all cases and all diagnostic categories, with no per-category optimization, in order to avoid data-driven overfitting. Only the resulting binary classification was recorded, and the per-indication probability values were not exported, so a threshold sensitivity analysis could not be performed retrospectively; this is acknowledged among the limitations. A different cutoff would be expected to shift the balance between sensitivity and specificity; a lower threshold would increase sensitivity at the expense of specificity and vice versa. Pathologies and the absence of pathologies related to tooth germs were not considered in the study.

### 2.4. Unit of Analysis and Diagnostic Categories

The unit of analysis was a single diagnostic indication, defined as the combination of one examination, one diagnostic category (Table 1), and one anatomical location, most often a specific tooth identified by its FDI number. Analysis at the level of the individual diagnostic indication, rather than at the level of the patient, was chosen because a single radiograph typically contains several independently assessable indications. Two levels of comparison were used and are reported separately. At the indication level (Table 2, Table 3 and Table 4), the software result and the reference standard had to agree on the diagnostic category as well as on the location. At the binary level (Table 5), each unit was classified only as pathology or no pathology, irrespective of the category assigned. A tooth on which the expert recorded caries and the software recorded an apical lesion is therefore discordant at the indication level but concordant at the binary level.

The following diagnostic categories were considered in the study: Caries/Crown Defect, Apical Pathology, Periodontal Bone Loss/Perio Widening, Endo-iatrogenic, Implant-Pathology, Tooth Position/Development, Fracture, Other Pathology, Maxillary Sinus Pathology, No Pathology, and Missing Tooth. Indications were classified according to the full set of 11 categories presented in Table 1. Table 2, Table 3 and Table 4 report every category that occurred in the given modality, in the reference standard, or in the software output; no category was omitted from the category-level analysis. Categories represented by a few indications are shown alongside the others, but their metrics are unstable and are interpreted as descriptive only. Maxillary sinus pathology was scored per sinus side rather than per tooth.

An indication was considered concordant when the AI result and the reference standard concerned the same imaging modality, the same case, the same diagnostic category, and the same anatomical location. A tooth at which more than one pathological condition was recorded contributed one indication for each of them, so the same tooth could yield a true positive in one category and a false negative in another. An indication assigned by the software to a different tooth from the one recorded in the reference standard was not counted as concordant: it was counted as a false negative for the reference tooth and, where the tooth to which it was assigned had been classified as free of pathology, as a false positive at that location. Where a reader recorded both a pathological indication and a “No pathology” label for the same location, the location was classified as positive for that reader because a pathological indication had been recorded there. Two such internal inconsistencies were present in the reference standard for panoramic radiographs; how they were resolved, by whom, and at what stage is set out in rule (10) of Section 2.5.

In the case of multifocal or extensive lesions, each location assigned to a separate tooth or structure was treated as a separate diagnostic indication.

A unit was classified as negative when the reader recorded a “No pathology” indication for that tooth or structure and recorded no pathological indication there. A negative result, therefore, required an explicit “No pathology” label rather than the mere absence of any report. Where the software returned no entry at all for a location that the expert had classified as free of pathology, the location was counted as a concordant negative because the software recorded no indication there. The set of negative units was thus determined by the charting performed by the expert and by the software, and not by a complete inventory of all teeth and anatomical sites defined in advance; the consequences of this are addressed in the Limitations. Missing teeth were not counted as negatives: the absence of a tooth was recorded as a separate diagnostic category (Missing Tooth) on the positive side of the matrix, and the absence of third molars was not treated as a missing tooth. No locations had to be excluded as non-evaluable, and no examination produced an indeterminate result for either the index test or the reference standard. At the level of the individual location, however, the software produced no entry at 23 of the locations charted by the expert. These are missing index test results in the sense of STARD items 15 and 16; how they were handled is stated in rule (6) of Section 2.5, and they are shown in Figure 1 and listed in Supplementary Material S2.

The category Caries/Crown Defect groups carious lesions together with crown defects, non-carious defects, and features of hard-tissue wear. This grouping reflects the way in which the indications were recorded in the source charting and could not be undone retrospectively because the underlying labels do not distinguish these subtypes. Statements about the detection of caries specifically must therefore be read with the composite nature of this category in mind.

The “No pathology” category was included as a separate diagnostic indication because, in clinical practice, not only the identification of pathology but also the correct recognition of the absence of changes in the assessed tooth or structure is important. In the present study, a concordant “No pathology” indication denoted a situation in which both the reference standard and the AI did not indicate any pathology at the same location. Such cases were treated as concordant diagnostic indications in the per-indication analysis and were considered true-negative results (TN) in the overall “pathology vs. no pathology” matrix; a full matrix of all possible categories and locations for each tooth was not constructed. False-positive results denoted indications generated by the AI that were not confirmed in the reference standard. False-negative results denoted indications present in the reference standard that the AI did not detect. Specificity and negative predictive value are reported only at the overall “pathology vs. no pathology” level because a category-specific true negative would require a complete negative universe that was not established; for the individual diagnostic categories, sensitivity, precision, and F1 are therefore reported.

### 2.5. Comparison of AI Results with Expert Assessment

The Diagnocat results were compared with the reference standard separately for intraoral, panoramic, and CBCT images. For each imaging type, the number of indications present in the reference standard, the number of indications generated by the AI, and the number of concordant and discordant indications were determined. The comparison was carried out at two levels. At the level of individual diagnostic categories, each indication was classified as a true positive (TP—agreement between the AI and the reference standard regarding category and location), a false negative (FN—an indication present in the reference standard but not detected by the AI), or a false positive (FP—an AI indication not confirmed in the reference standard).

The following counting and classification rules were applied throughout and are stated in full so that the analysis can be reproduced from the source records.

(1) Two analyses were performed. The per-indication analysis (Table 2, Table 3 and Table 4) is the primary analysis of this study. The binary “pathology versus no pathology” analysis (Table 5) is secondary and exploratory, and the specificity, NPV, and accuracy derived from it are reported as secondary descriptive outcomes.
(2) Unit of observation. In the per-indication analysis, the unit is a single diagnostic indication, defined as the combination of one examination, one diagnostic category, and one anatomical location. In the binary analysis, the unit is one anatomical location, that is, one tooth, one named non-dental structure such as a maxillary sinus, or the examination as a whole where an indication was recorded without a specific location. The term “diagnostic indication” is used throughout for a single recorded entry and the term “location” for the tooth or structure to which it refers; the two terms are not used interchangeably. The totals of the two analyses, therefore, differ and are not expected to coincide.
(3) Analysis set. The locations entering the binary analysis are those charted by the expert. Every examination was charted by the expert before the software output was consulted, and the software output did not influence which locations were included.
(4) Status of a location. A location is positive for a given reader if that reader recorded at least one pathological indication there and negative if that reader recorded only a “No pathology” indication. A tooth carrying several pathological indications is therefore one positive location in the binary analysis and several separate indications in the per-indication analysis.
(5) Indications recorded without a location. Five indications, all in the Other Pathology category, were recorded without a specific tooth or structure: two in the intraoral set and two in the panoramic set by the expert and one in the panoramic set by the software. Each was treated as a single examination-level location, so that such an indication could be matched between readers within the same examination. In one panoramic examination, both readers recorded an examination-level indication, and that location was counted as a true positive; the remaining three expert indications of this kind were counted as false negatives.
(6) Locations charted by the expert for which the software produced no entry. The software does not report on every anatomical location, so the absence of an entry does not mean that the location was assessed and found normal. This occurred at 23 locations in total: 3 intraoral, 10 panoramic, and 10 CBCT. They constitute missing index test results at the level of the individual location, in the sense of STARD items 15 and 16, and are reported as such in Figure 1. In total, 22 of them were positive in the reference standard and were counted as false negatives, which is the conservative choice and counts against the software; one, a third molar recorded as free of pathology, was counted as a true negative. Excluding that single location changes no reported value to two decimal places. All 23 locations are listed in Supplementary Material S2, together with a sensitivity analysis in which all of them are excluded.
(7) False negatives, therefore, arise in two distinct ways: at locations where the software produced no entry at all (22 locations, as above) and at locations that the software charted but classified as free of pathology (65 locations). The two groups are reported separately in Supplementary Material S2.
(8) Locations reported by the software but not charted by the expert. One intraoral, two panoramic, and eight CBCT locations were reported by the software at locations for which the reference standard provides no status. These locations were excluded from the binary analysis because they can be classified neither as correct nor as incorrect against the reference standard, and they are listed individually in Supplementary Material S2.
(9) Indications assigned to a different location. An indication recorded by the software at a tooth other than the one recorded in the reference standard was not counted as concordant.
(10) Conflicting labels. Where a reader recorded both a pathological indication and a “No pathology” label for the same location, the location was classified as positive for that reader. Two such conflicts occurred in the panoramic reference standard, at tooth 12 of examination F1 and at tooth 34 of examination F4. These two were not settled by the rule but individually, by the first author (W.C.), against the expert’s original source records for those examinations; the radiographs themselves were not reread for this purpose. This was done during the revision of the manuscript, at a stage when the software output was already available to that author. The resolution did not follow the software: tooth 12 was resolved as free of pathology, although the software had recorded caries at that location, so the decision counts against the software rather than in its favour. The expert who established the reference standard took no part in this step and remained blinded to the software output throughout. Three conflicts of the same kind occurred in the software output, at tooth 12 of panoramic examination F1, tooth 46 of panoramic examination M3, and tooth 18 of CBCT examination M1. The index test output was analysed exactly as produced, and all three locations were classified as positive under the rule stated above, without exception.
(11) Missing teeth were recorded as a separate diagnostic category on the positive side of the matrix. The absence of a third molar was not counted as a missing tooth; this exclusion applies only to the Missing Tooth category, and other indications recorded on third molars, such as abnormalities of position or development, were analysed in the same way as indications on other teeth.
(12) Data verification. During the verification of the source records carried out in response to the reviewers, the edentulous areas described in the reference report of one CBCT examination (F8) were found not to have been transcribed tooth by tooth into the structured dataset. The corresponding 11 missing-tooth indications were completed by the first author (W.C.) from the written reference report for that examination, together with the teeth the expert had charted as present. The software output was not used to derive them, but it was already available to that author at the time, and the 11 FDI numbers so derived coincide exactly with those reported by the software for that examination. Because this correction increases the agreement between the two readers, the CBCT values obtained before it was applied are reported in Section 3.5 as a sensitivity analysis. All other examinations were checked for untranscribed edentulous areas, and none were found; the missing-tooth counts of both readers for each of the 15 CBCT examinations are given in Supplementary Material S2, as is the full effect of the correction.

**Figure 2:**
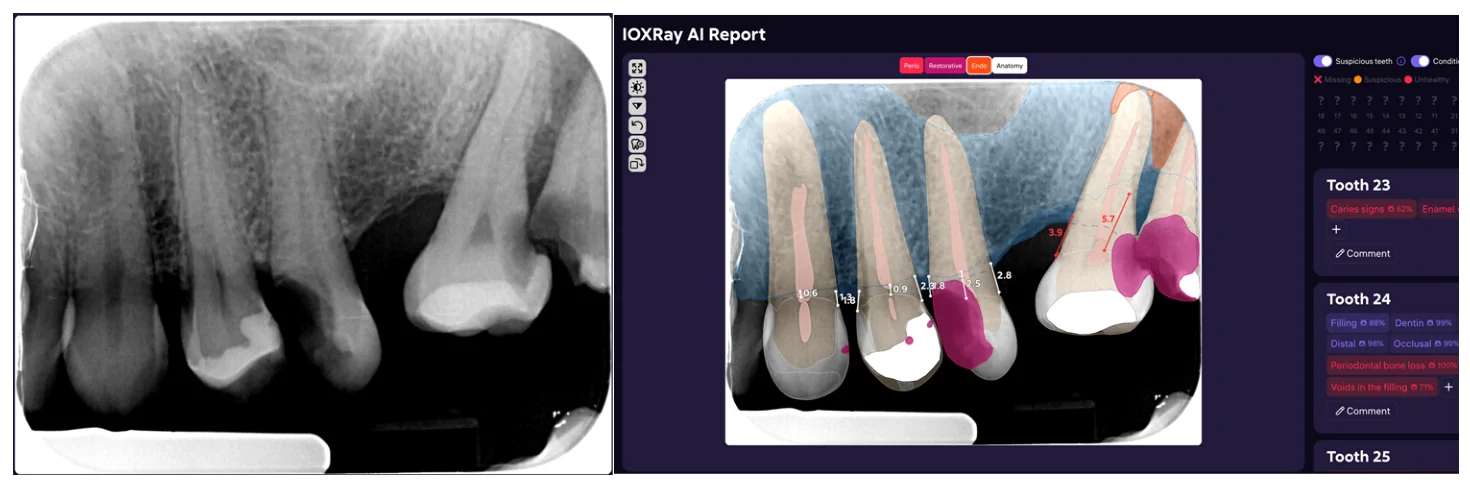
On the **left**, an example intraoral radiograph in the form provided to the expert for assessment. On the **right**, an example report generated by the Diagnocat system for the same image. Results were compared at the level of each individual diagnostic indication, taking into account the category of the change and its location within a specific tooth.

**Figure 3:**
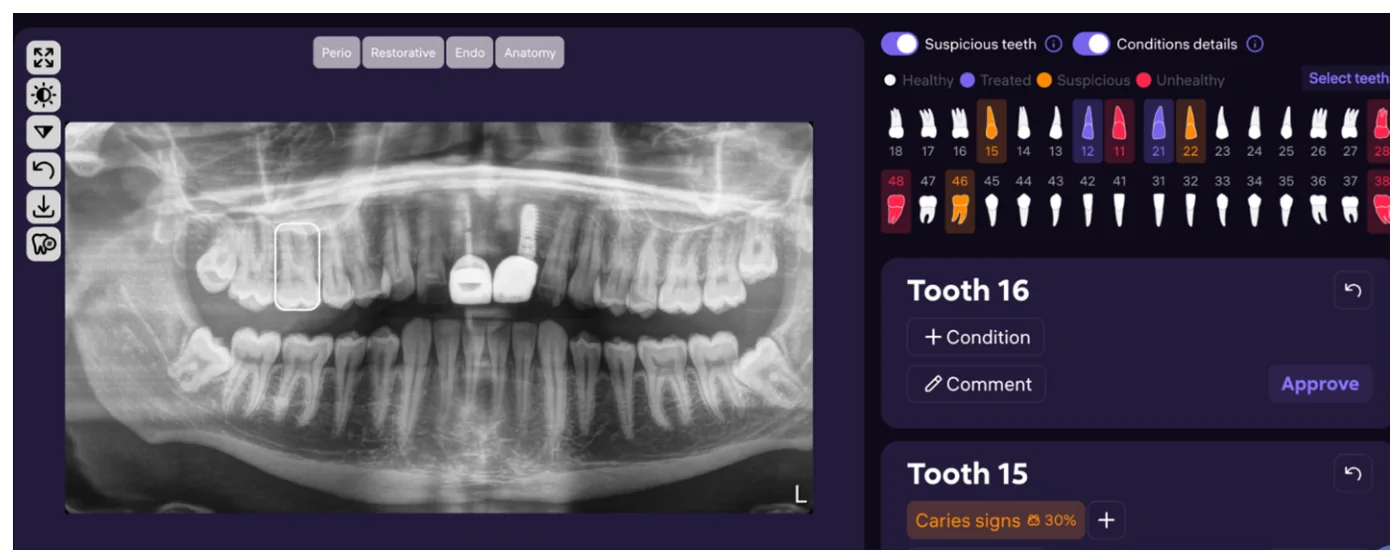
Example of correct marking of the absence of pathology on a panoramic radiograph. Both the expert creating the reference standard and the Diagnocat software found no pathological changes within the marked tooth. Cases of this type were treated in the analysis as concordant diagnostic indications concerning the absence of pathology.

At the overall level, a four-field “pathology vs. no pathology” matrix was constructed, in which TP denoted a concordant pathological indication, FN a pathology present in the reference standard and not detected by the AI, FP a tooth without pathology in the reference standard to which the AI assigned a change, and TN a concordant “No pathology” indication, i.e., an absence of pathology stated simultaneously by the reference standard and the AI at the same location. “Missing tooth” indications were counted on the pathology side. An example of an intraoral radiograph as provided to the expert, together with the corresponding report generated by the software, is shown in Figure 2. An example of a location at which both readers recorded the absence of pathology, and which therefore contributed a true negative, is shown in Figure 3.

As in the studies by Orhan et al. [12] and Turosz et al. [6], the unit of analysis was referred to the level of a single tooth/indication rather than the whole image; the adopted method of assessing agreement using a four-field table was also consistent with earlier validations of the Diagnocat software [9,10,11] and with the manner of reporting diagnostic measures used by Kazimierczak et al. [13] and Issa et al. [14]. In parallel, a separate stage of the study was conducted involving the assessment of the same images by dentists; however, that analysis is not the subject of the present article. Because the Missing Tooth category accounted for a substantial share of all indications, particularly in CBCT, the binary analysis was additionally repeated with this category excluded, as a sensitivity analysis. Because the two analyses use different units of observation, their totals differ and are not expected to coincide: the per-indication analysis counts every recorded indication, whereas the binary analysis counts each tooth or anatomical site once. A false positive in the per-indication analysis also includes a software indication recorded at a location where the reference standard recorded a different pathology, and a software indication recorded at a location for which the reference standard records no status at all; there were 11 locations of the latter kind, one intraoral, two panoramic, and eight CBCT, and they are listed in Supplementary Material S2. A false positive in the binary analysis, by contrast, is counted only at a location classified as free of pathology; the false-positive counts in Table 2, Table 3 and Table 4 are therefore higher than those in Table 5. Supplementary Material S2 sets out, for each modality, how the counts of diagnostic indications reported in the Results relate to the binary matrix in Table 5.

### 2.6. Statistical Analysis

A Microsoft Excel (Microsoft 365, version 2605; Microsoft Corporation, Redmond, WA, USA) spreadsheet was used for data management, in which a database was created and the indications recorded by the expert and by the Diagnocat software were matched. Categorical variables were described using counts and percentages. The following diagnostic measures were calculated: sensitivity [TP/(TP + FN)], specificity [TN/(TN + FP)], positive predictive value (PPV) [TP/(TP + FP)], negative predictive value (NPV) [TN/(TN + FN)], accuracy [(TP + TN)/(TP + FP + FN + TN)], and F1-score [2TP/(2TP + FP + FN)]. The analysis is descriptive throughout. No hypothesis tests were performed, and no confidence intervals are reported. The reason is the clustered structure of the data: each examination contributed many diagnostic indications, so indications originating from the same image and the same patient are not independent. With 15 examinations per modality, methods that assume independent observations, such as the Pearson χ^2^ test and Wilson intervals, would produce intervals that are too narrow and *p*-values that are too small, while resampling patients as clusters would not yield informative precision at this sample size. Point estimates are therefore reported without measures of precision, and all comparisons between modalities and between diagnostic categories are presented as numerical differences observed in this sample rather than as statistical inferences.

## 3. Results

### 3.1. Characterization of the Material and Number of Diagnostic Indications

A total of 45 radiological examinations were included in the analysis, comprising 15 intraoral radiographs, 15 panoramic radiographs, and 15 CBCT examinations. Both pathological indications and “No pathology” indications, treated as full-fledged diagnostic units, were considered.

Overall, 999 diagnostic indications were recorded in the reference expert assessment, whereas the AI-based software recorded a total of 1017 indications. The smallest number of indications was recorded for intraoral radiographs, along with a markedly larger number for panoramic and CBCT examinations. This resulted from the greater range of assessed structures and the larger number of potential diagnostic observations in these imaging types.

For intraoral radiographs, the number of diagnostic indications in the reference standard was 71 and 69 in the AI analysis. For panoramic radiographs, 452 and 444 indications were recorded, respectively, whereas for CBCT, there were 476 and 504 indications.

In the pooled analysis covering all modalities, 791 concordant diagnostic indications were obtained, with 208 indications present in the reference standard that the AI did not detect and 226 indications generated by the AI without confirmation in the expert assessment. In the secondary binary analysis, in which the unit is one tooth or anatomical site, 775 of 944 locations were classified concordantly, corresponding to an overall agreement of 82.1%. Detailed values for the individual modalities are presented below. These results should be regarded as a general characterization of the system’s performance across the whole material because AI performance differed between the individual imaging modalities and diagnostic categories.

### 3.2. Results for Intraoral Radiographs

For intraoral radiographs, the reference standard contained 71 diagnostic indications and 69 indications in the AI analysis. The number of concordant indications was 45, the number of indications present in the reference standard but not detected by the AI was 26, and the number of indications generated by the AI without confirmation in the expert assessment was 24.

In the secondary binary analysis, sensitivity was 0.79, specificity 0.59, precision (PPV) 0.71, NPV 0.70, accuracy 0.70, and F1-score 0.75 (TP 27, FN 7, FP 11, TN 16, over 61 locations).

A more detailed analysis revealed numerical differences between diagnostic categories. The highest values were recorded for the Endo-iatrogenic category (sensitivity 1.00, precision 0.88, F1 = 0.93), as well as for the Missing Tooth category (sensitivity 1.00, precision 0.75, F1 = 0.86), and Apical Pathology (sensitivity 0.80, precision 0.67, F1 = 0.73). Intermediate values were recorded for the Caries/Crown Defect category (sensitivity 0.70, precision 0.64, F1 = 0.67). The lowest F1 value among the more frequently represented categories was obtained for Periodontal Bone Loss/Perio Widening. Categories such as Fracture, Tooth Position/Development, and Other Pathology were represented by single or a few indications; their results are reported descriptively and do not support any assessment of the system’s performance in those categories.

### 3.3. Results for Panoramic Radiographs

For panoramic radiographs, the reference standard contained 452 diagnostic indications and 444 indications in the AI analysis. The number of concordant indications was 358, the number present in the reference standard but not detected by the AI was 94, and the number generated by the AI without confirmation in the expert assessment was 86.

In the secondary binary analysis, sensitivity was 0.73, specificity 0.88, precision (PPV) 0.79, NPV 0.84, accuracy 0.82, and F1-score 0.75 (TP 117, FN 44, FP 32, TN 236, over 429 locations).

The analysis of the distribution of diagnostic categories revealed numerical differences between the individual types of indications. The highest values were recorded for the Missing Tooth category (sensitivity 0.94, precision 0.88, F1 = 0.91). Higher values were also recorded for the Endo-iatrogenic category (sensitivity 0.74, precision 0.78, F1 = 0.76) and Tooth Position/Development, for which precision was 0.85 at a sensitivity of 0.61 and F1 = 0.71. Lower values were recorded for the Apical Pathology category (sensitivity 0.67, precision 0.67, F1 = 0.67) and Periodontal Bone Loss/Perio Widening (sensitivity 0.69, precision 0.69, F1 = 0.69). The lowest values were recorded for the Caries/Crown Defect category, for which the system achieved sensitivity 0.59, precision 0.52, and F1 = 0.55, as well as the Other Pathology (sensitivity 0.25, precision 1.00, F1 = 0.40) and Maxillary Sinus Pathology (sensitivity 0.33, precision 1.00, F1 = 0.50) categories. For these last two categories, the AI generated a few false-positive indications but did not detect a substantial proportion of the changes present in the reference standard.

### 3.4. Results for CBCT Examinations

For CBCT examinations, the reference standard contained 476 diagnostic indications and 504 indications in the AI analysis. The number of concordant indications reached 388, the number present in the reference standard but not detected by the AI was 88, and the number generated by the AI without confirmation in the expert assessment was 116.

In the secondary binary analysis, sensitivity was 0.80, specificity 0.86, precision (PPV) 0.78, NPV 0.87, accuracy 0.83, and F1-score 0.79 (TP 141, FN 36, FP 39, TN 238, over 454 locations). These were the numerically highest sensitivity and F1-score among the analyzed imaging types.

A more detailed analysis revealed numerical differences between diagnostic categories. The highest values were recorded for the Endo-iatrogenic category (sensitivity 0.91, precision 0.91, F1 = 0.91), as well as for Maxillary Sinus Pathology (sensitivity 0.85, precision 0.85, F1 = 0.85). Higher values were also recorded for the Tooth Position/Development category (sensitivity 0.71, precision 0.83, F1 = 0.77). For the Apical Pathology category, the system achieved high sensitivity (0.84) but markedly lower precision (0.53), indicating a substantial number of false-positive indications. A similar result was observed for the Caries/Crown Defect category, for which sensitivity was 1.00, precision 0.34, and F1 = 0.51. The lowest values were recorded for the Periodontal Bone Loss/Perio Widening category (sensitivity 0.83, precision 0.25, F1 = 0.38); however, the reference standard included only six indications in this category. A single-implant pathology indication was generated by the software and was not confirmed by the reference standard. These results are reported as qualitative observations in a small sample rather than as an assessment of the system’s performance in these categories.

### 3.5. Comparison of Results Between Imaging Types

The results for the three types of radiographs are summarized together in Table 5. In the secondary binary analysis, sensitivity was 0.79 for intraoral radiographs, 0.73 for panoramic radiographs, and 0.80 for CBCT, and the F1-score was 0.75, 0.75, and 0.79, respectively; sensitivity and F1-score were numerically highest for CBCT. Specificity, NPV and accuracy are not compared between the modalities because the set of negative units followed from the locations charted by the expert and differs in size and composition between the three groups.

A summary of the secondary binary analysis for all three modalities is presented in Table 5. No formal statistical comparison between the modalities was performed, for the reasons given in Section 2.6, and the differences described above are numerical differences observed in this sample. Because the three groups comprised different patients, different anatomical regions, and different distributions of diagnostic categories, they cannot be read as evidence that any modality is intrinsically superior.

A sensitivity analysis was performed in which the Missing Tooth category was removed from the binary matrix. For panoramic radiographs, the values changed from sensitivity 0.73, specificity 0.88, accuracy 0.82, and F1-score 0.75 to 0.66, 0.89, 0.81, and 0.70, respectively (TP 86, FN 45, FP 29, TN 239). For CBCT, they changed from 0.80, 0.86, 0.83, and 0.79 to 0.84, 0.87, 0.86, and 0.75, respectively (TP 77, FN 15, FP 35, TN 242). For intraoral radiographs, they changed from 0.79, 0.59, 0.70, and 0.75 to 0.77, 0.59, 0.69, and 0.73 (TP 24, FN 7, FP 11, TN 16). Removing the category, therefore, lowered sensitivity for panoramic radiographs and raised it for CBCT; the other measures moved in the same direction for both modalities. The full derivation is given in Supplementary Material S2.

A second sensitivity analysis concerns the data correction described in rule (12) of Section 2.5. Before the 11 missing-tooth indications were completed in the reference standard of CBCT examination F8, the CBCT reference standard contained 465 diagnostic indications, and the binary matrix gave sensitivity 0.78, specificity 0.86, precision (PPV) 0.77, NPV 0.87, accuracy 0.83, and F1-score 0.78 (TP 130, FN 36, FP 39, TN 238, over 443 locations). In the Missing Tooth category, the reference standard then held 76 indications against 64 recorded by the software, with 49 true positives, 27 false negatives, and 15 false positives; after the correction, the same row reads 87 and 64, with 60 true positives, 27 false negatives, and 4 false positives. The correction, therefore, raises CBCT sensitivity from 0.78 to 0.80 and the F1-score from 0.78 to 0.79 and leaves specificity, NPV, and accuracy unchanged to two decimal places. The correction was made with knowledge of the software output, as stated in Section 2.5, and both sets of values are reported so that their effect is visible.

## 4. Discussion

In the present study, the diagnostic accuracy of the Diagnocat software depended both on the imaging modality and on the type of diagnostic indication assessed. The highest sensitivity and F1-score were recorded for CBCT and the lowest F1-score for panoramic radiographs and intraoral radiographs, which were equal. Specificity, NPV, and accuracy are not compared between the modalities, for the reason given in the Limitations. Direct comparison with other studies is limited by differences in study design, unit of analysis, and definition of endpoints, and the differences observed here should be read as descriptive observations in a pilot sample.

The dependence of the AI result on the modality was well demonstrated by Kazimierczak et al. [13], who assessed the performance of Diagnocat in detecting periapical lesions on panoramic radiographs and CBCT. In their study, AI sensitivity for panoramic radiographs was 33.33% and F1 32.73%, whereas for CBCT, sensitivity increased to 77.78% and F1 to 84.00%, with specificity above 98% in both modalities. In our material, CBCT likewise showed the highest sensitivity.

For intraoral radiographs, the more frequently represented categories, Caries/Crown Defect, Endo-iatrogenic, and Apical Pathology, allow the most reliable observations; Endo-iatrogenic and Apical Pathology were relatively better recognized, whereas Caries/Crown Defect was moderate. Ibraheem et al. [5], assessing AI Second Opinion software on periapical radiographs, reported high sensitivity for many categories, including 91.0% for caries, 86.6% for periapical lesions, 93.4% for endodontic treatment, and 91.1% for marginal bone loss. Our intraoral values for precision and overall agreement were lower, but the same dependence of the result on the diagnostic category was present, which indicates that an AI system on intraoral radiographs should not be judged by a single global value.

In the context of caries diagnosis on intraoral radiographs, the study by Szabó et al. [10] is also important, in which the use of AI in recognizing carious lesions on intraoral radiographs was assessed. The authors pointed out that one of the important limitations of this type of assessment remains the lack of direct clinical validation. In our material, the Caries/Crown Defect category on intraoral radiographs achieved a moderate result, which supports the position that a positive caries indication by an AI system should be treated as a signal requiring clinical verification, rather than as a standalone basis for a therapeutic decision.

For the interpretation of intraoral results, the study by Allihaibi et al. [15] is also important, in which the Diagnocat platform was assessed on periapical radiographs, with CBCT adopted as the reference standard. At the tooth level, the AI achieved higher sensitivity than the clinicians, 67.3% versus 49.3%, but lower specificity, 82.3% versus 92.5%. At the root level, a similar profile was observed: AI sensitivity was 54.3% versus 43.8% for clinicians and specificity 86.7% versus 94.5%. In our intraoral material, a similar problem was visible, especially for Caries/Crown Defect.

With regard to periapical lesions on two-dimensional radiographs, the study by Issa et al. [14] can also be cited, in which Diagnocat achieved sensitivity 92.30%, specificity 97.87%, accuracy 96.66%, and F1 0.92 in detecting changes in the periapical tissues on 2D radiographs. In our study, the Apical Pathology category on intraoral radiographs was also among the better-recognized, although the metric values were lower.

In the analysis of panoramic radiographs, Diagnocat achieved sensitivity 0.73, specificity 0.88, precision 0.79, and F1-score 0.75. A very good point of reference is the study by Mema et al. [16], in which 104 panoramic radiographs were analyzed using Diagnocat, and the results were compared with the assessment of three experts. The authors obtained a very high overall system sensitivity, 99.2%, but a very low specificity, 8.7%, indicating a tendency of the system toward overdiagnosis. Cohen’s κ coefficient was higher for missing teeth and fillings, κ = 0.37 and κ = 0.39, respectively, than for caries and periodontal bone loss, where it took negative values, κ = −0.15 and κ = −0.62, respectively. This is consistent with our results: Missing Tooth on panoramic radiographs was one of the strongest categories, whereas Caries/Crown Defect was clearly weaker.

Detailed data from the study by Mema et al. [16] additionally reinforce the interpretation of our panoramic results. For caries, Diagnocat achieved an AUC of 0.73, sensitivity 0.95, and specificity 0.47, indicating high detectability of positive cases with a limited ability to correctly exclude cases without features of caries. For periodontal bone loss, an AUC of 0.85, sensitivity 0.96, and specificity 0.71 were obtained; however, the system often indicated periodontal bone loss in places where the experts did not confirm it. In our panoramic material, the Periodontal Bone Loss/Perio Widening category was more balanced than in the study by Mema et al., with sensitivity and precision both 0.69, but Caries/Crown Defect retained a similar problem: the system simultaneously overlooked some indications and generated a substantial number of additional indications.

It is also worth citing the study by Kwiatek et al. [11], in which Diagnocat was assessed in detecting caries on panoramic radiographs compared with the clinical assessment of dentists. The authors showed that the agreement of the system with the physicians’ assessment was variable and depended, among others, on the tooth location, with the lowest agreement observed in the premolar region.

Similar observations follow from the study by Orhan et al. [12], in which Diagnocat was assessed on 100 panoramic radiographs comprising 4497 teeth. Diagnocat and three maxillofacial radiologists assessed a wide spectrum of radiological conditions, including caries, fillings, loss of contact points, periapical lesions, periodontal bone loss, implants, crowns, missing teeth, and features of endodontic treatment. The authors demonstrated high system sensitivity for categories with a more unambiguous radiological image, such as absence of pathology, prosthetic crowns, calculus, missing teeth, fillings, absence of a contact point, periodontal bone loss, and implants. At the same time, low sensitivity was recorded for caries, periapical lesions, pontics, deficiencies in root canal fillings, and overhangs.

It is worth referring separately to endodontic indications. In our material, Endo-iatrogenic was among the better-recognized categories across intraoral, panoramic, and CBCT imaging. Kazimierczak et al. [17] assessed Diagnocat on 55 panoramic radiographs with respect to endodontic treatment outcomes. The system achieved high accuracy, 90.72%, and F1, 95.12%, in detecting the probable presence of an endodontic filling, but markedly worse results for more qualitative features of treatment: F1 was 14.29% for deficiencies in the filling and 8.33% for underfilling of the canal. This distinction is important for our interpretation: AI recognizes the presence of distinct features, such as endodontic material, well, but may have greater difficulty with a more detailed assessment of treatment quality. In our study, the Endo-iatrogenic category comprised various endodontic complications and artifacts; therefore, the high results should be interpreted as a favorable signal, but not as a full validation of the system for all detailed aspects of root canal treatment.

**Figure 4:**
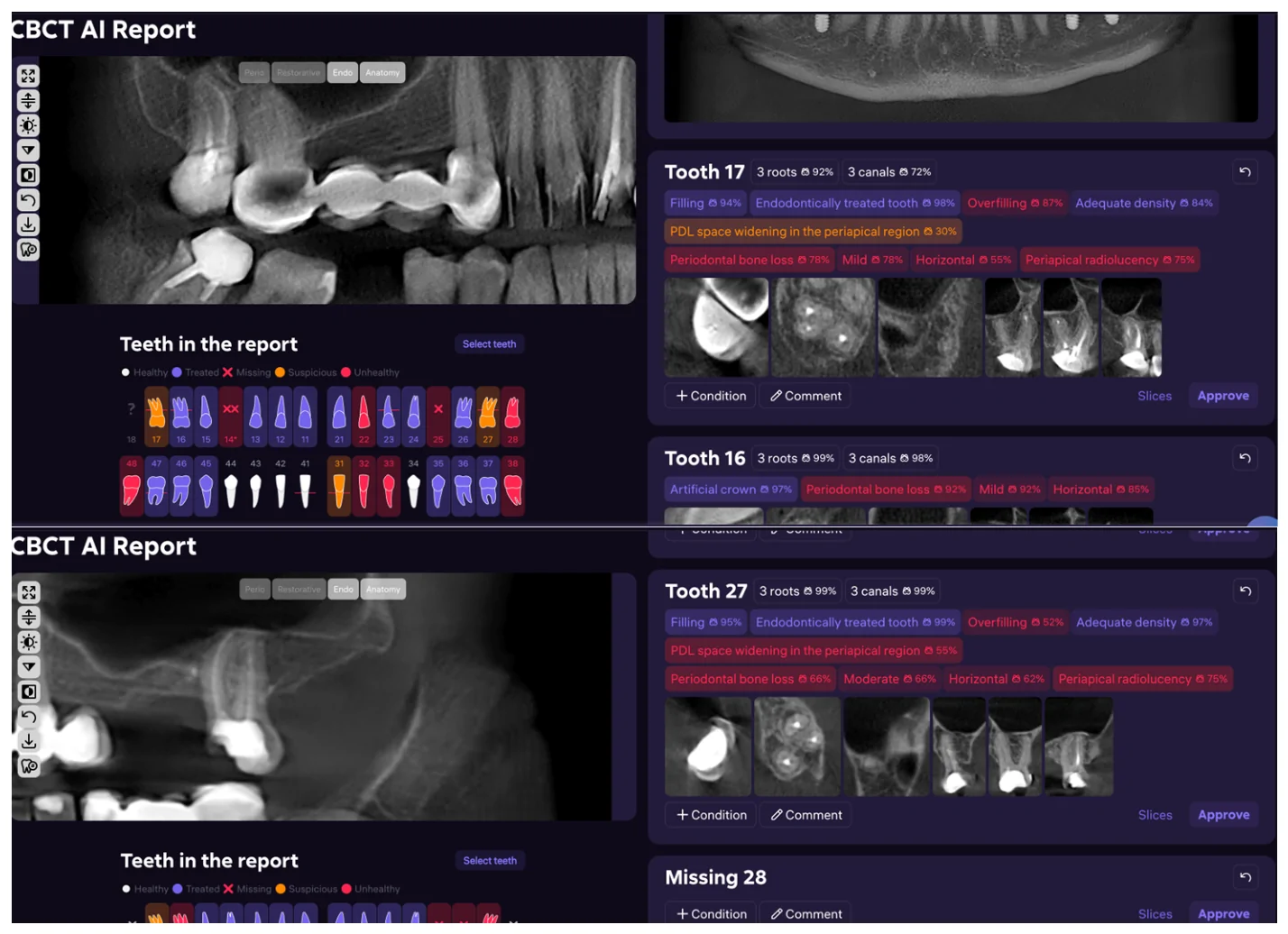
Examples of false-positive results generated by the Diagnocat system on CBCT. In the presented cases, the system marked, among others, overfilling and PDL space widening in the periapical region. In the expert assessment, these indications were not confirmed, and the image corresponded to a normal anatomical structure in the vicinity of the maxillary sinus floor, erroneously interpreted by the AI as a pathological change. These examples illustrate the necessity of verifying AI-generated indications in the context of a complete radiological assessment.

In CBCT examinations, Diagnocat achieved the highest sensitivity among all modalities but, at the same time, generated the most additional indications not confirmed by the reference standard. Examples of such false-positive indications on CBCT are shown in Figure 4. The best results concerned Endo-iatrogenic and Maxillary Sinus Pathology. In turn, the greatest problems with precision were observed in the Periodontal Bone Loss/Perio Widening, Caries/Crown Defect, Other Pathology, and Apical Pathology categories. For Periodontal Bone Loss/Perio Widening and Caries/Crown Defect, the number of indications in the reference standard was limited; therefore, conclusions concerning these categories should be cautious. Across modalities, false-positive indications were concentrated in the categories with the lowest precision, in particular, Other Pathology, Caries/Crown Defect, and Periodontal Bone Loss on CBCT, whereas false negatives were most frequent for Missing Tooth on CBCT and for Caries/Crown Defect on panoramic radiographs; this indicates a tendency toward overcalling on volumetric imaging and underdetection of subtle two-dimensional findings.

The CBCT results can be compared with the study by Ezhov et al. [9], in which the performance of the Diagnocat system for CBCT examinations was assessed. The authors used 1346 CBCT scans to train the system modules, and in the clinical part, 24 dentists assessed 30 CBCT examinations in an AI-supported group or without support. The group supported by Diagnocat achieved higher sensitivity than the group without AI support, 0.8537 versus 0.7672, with very similar specificity, 0.9672 versus 0.9616.

Our results for Apical Pathology on CBCT are worth relating to the study by Allihaibi et al. [7], in which Diagnocat was assessed in detecting periapical radiolucencies on CBCT scans of molars. The study indicates that Diagnocat achieved high sensitivity in detecting periapical lesions on CBCT, but with moderate specificity, which was associated with a risk of false-positive results. In our study, for the Apical Pathology category on CBCT, sensitivity was 0.84 and precision 0.53.

The results concerning the Maxillary Sinus Pathology category should be interpreted in the context of the imaging modality. In our material, on panoramic radiographs, the system showed low sensitivity with high precision, whereas on CBCT, it obtained a markedly better result. This may result from the nature of the imaging: assessment of the maxillary sinus on panoramic radiographs is limited by the superimposition of structures and the projective character of the image, whereas CBCT allows a three-dimensional analysis. The review by Uthman et al. [18] indicates that AI models used in CT/CBCT achieved high values of accuracy, sensitivity, and specificity in detecting maxillary sinus pathology; however, the range of results differed between studies and depended on the type of assessed pathology, the input data, and the reference standard. Shujaat et al. [19] also indicated the promising potential of AI in automating tasks related to the maxillary sinus in CBCT, especially in segmentation, classification, and implant planning, but emphasized the need for larger, more diverse datasets and standardization of reporting. In this context, our results fit the observation that CBCT may be a more advantageous modality for the assessment of the maxillary sinus, but they do not eliminate the need for further validation of AI systems in this category.

The present study also assessed the correct recognition of the absence of pathology, which corresponds to true-negative results in the binary matrix. Many studies evaluate AI only in terms of detecting positive findings, and the study by Mema et al. [16], in which very high sensitivity was accompanied by very low specificity, illustrates why the negative side matters. It must be stressed, however, that the set of negative units here was not enumerated in advance, as every tooth and location at which pathology could have been present. The true-negative count, and therefore specificity, NPV, and accuracy, depend on that operational definition and may be over- or underestimated. These measures should be read as internally consistent within the present framework, and not as absolute values or as a basis for comparison between modalities.

From a clinical point of view, the results support treating Diagnocat as a supporting tool rather than a standalone replacement for expert assessment. Pul et al. [20] demonstrated that AI support may influence physicians’ diagnostic decisions in the assessment of periapical lesions, and Ezhov et al. [9] showed that Diagnocat support in CBCT increased the sensitivity of physicians’ assessments without a marked deterioration in specificity. Mema et al. [16], in turn, demonstrated that high Diagnocat sensitivity may be accompanied by low specificity and a tendency toward overdiagnosis. In our material, a similar pattern was seen in CBCT, where high sensitivity was associated with a larger number of additional indications. Even where the overall agreement was high, the discordance between the AI and the expert was substantial: for intraoral radiographs, 45 of 71 reference indications were concordant, with 26 not detected and 24 added by the AI; for panoramic radiographs, 94 of 452 reference indications were not detected, and 85 were added; and for CBCT, 88 of 476 were not detected, and 116 were added. Roughly one in five reference indications on panoramic and CBCT examinations were therefore not reproduced by the software, and a substantial number of additional indications were generated, so that in its current form, the system requires correction by a clinician before any therapeutic decision is made.

The study has several limitations. First, it is of a pilot nature and includes a limited number of examinations in each modality. Second, the reference standard was established by a single expert. This is the principal limitation of the study: extensive clinical experience does not by itself guarantee a valid reference standard, and every performance measure reported here may have been affected by that reader’s systematic classification tendencies, in either direction. The absence of a second, independent reader blinded to the AI output precluded any assessment of inter-observer agreement, and because the examinations were not reread by the same expert after an interval, intra-observer reliability could not be assessed either.

Third, the unit of analysis was a single diagnostic indication rather than the patient, so many observations originated from the same image and are not independent. Mema et al. [16] pointed to a similar problem in tooth-level analysis: treating many teeth from the same patient as independent observations may lead to overestimation of the certainty of diagnostic metrics. For this reason, the analysis was restricted to descriptive statistics, and no confidence intervals or hypothesis tests are reported; a formal clustered model was not fitted because it would not yield informative estimates with 15 examinations per modality. Fourth, the set of negative units was not defined a priori as a complete inventory of all teeth and anatomical sites, but follows from the indications recorded by the expert. The number of true negatives, and therefore specificity, NPV, and accuracy, are conditional on that operational definition and would change under a different one; these three measures should not be compared across modalities on the basis of the present data.

Fifth, the acquisition parameters of the individual examinations, including device models, the intraoral image receptor, the panoramic acquisition settings, and the CBCT field of view and voxel size, could not be retrieved from the anonymized archive and are therefore not reported. Because the software and the expert assessed exactly the same image files, this omission cannot bias the comparison between the index test and the reference standard, but it does limit the transferability of the results to other equipment. Accordingly, the findings should not be generalized to other devices, acquisition protocols, or clinical settings. Sixth, although both centers contributed material to all three modalities, the numerical distribution of examinations between centers was not recorded at the time of anonymization and could not be reconstructed. Seventh, the examinations were selected in a non-systematic (convenience) manner by an investigator who took no part in the reference-standard assessment, so selection bias cannot be excluded. Eighth, only one AI system (Diagnocat) was evaluated, and a single decision threshold of 50% was applied, chosen a priori by the investigators; no threshold optimization and no ROC analysis were performed. Finally, the grading of periodontal lesions as moderate or severe relied on qualitative assessment rather than on measurement of the proportion of root length affected, and the correspondence between the internal grading of the software and established radiographic staging systems was not verified. The findings should be regarded as exploratory, hypothesis-generating, and feasibility-oriented, with no claim of clinical generalizability.

A further limitation is the absence of the patient’s clinical data. Both Diagnocat and the expert assessed the images without access to symptoms, treatment history, clinical examination, or risk factors. This made it possible to compare pure radiological interpretation, but does not fully reflect the actual diagnostic process. This is particularly important in caries, periodontal diseases, and periapical lesions, where the final clinical decision requires combining the radiological image with an examination of the patient.

Another limitation was the use of intraoral and panoramic radiographs in JPEG format. This format was applied deliberately because, in parallel, an analysis of the same diagnostic material was conducted by dentists and by the AI system, and the aim was to ensure identical input data for all assessed groups. In addition, JPEG remains one of the most frequently used formats for exporting 2D images in everyday clinical practice and in communication between systems. It should be taken into account, however, that image compression and the manner of export may affect the visibility of subtle radiological changes, particularly in the case of small carious lesions, marginal bone loss, or minor periapical changes. The degree of JPEG compression was not quantified.

In the review by Mohammad-Rahimi et al. [21] concerning the use of AI in caries detection, it was emphasized that the available studies differ in the type of analyzed images, the manner of defining carious lesions, and the adopted methods of assessing performance. Similarly, Patil et al. [22], analyzing the use of AI in assessing periodontal bone loss, drew attention to the diversity of available algorithms, the limited quality of some studies, and the need for further clinical validation. Therefore, the obtained results should be treated as a radiological assessment of individual diagnostic indications, and not as a complete clinical diagnosis of the patient.

Further studies should include assessment by at least two independent readers blinded to the AI output, which would permit evaluation of inter-observer agreement, and repeated reading by the same expert after an interval, which would permit evaluation of intra-observer reliability. Larger and more diverse datasets, analysis of diagnostic categories in adequately sized subgroups, and a direct comparison of AI results with the assessment of dentists are also needed. In the present material, the system worked most consistently in categories with a more unambiguous radiological appearance, whereas the greatest limitations concerned caries, periodontal changes, non-specific changes, and some periapical indications. Whether the software could serve as a screening tool, as a second diagnostic opinion, or as an element supporting the standardization of radiographic reporting cannot be determined from this study because the performance of clinicians with and without AI assistance was not compared. These remain hypotheses for future work. This is consistent with the conclusions of Heo et al. [23], who emphasize that the practical implementation of AI in oral and maxillofacial radiology requires not only high diagnostic metric values, but also large, well-described datasets, consistent reference standards, and the participation of experienced radiologists in the validation process.

## 5. Conclusions

In this pilot study, the agreement between Diagnocat and a single-expert reference standard varied by imaging modality and by diagnostic category, and performance was weakest for subtle two-dimensional findings. Because the analysis was descriptive and the set of negative units followed from the expert’s charting rather than from a predefined anatomical inventory, no comparison between modalities can be claimed as an established difference. Within its exploratory scope, the study suggests that Diagnocat may support, but should not replace, expert interpretation of dental radiographs.

## Figures and Tables

**Table 1:** Operational definitions of the diagnostic categories included in the analysis.

Category	Operational Definition
Caries/Crown Defect	Radiological features of caries or a crown defect at the tooth level, without distinguishing between surfaces. The category also included non-carious defects and features of wear of the hard tooth tissues.
Apical Pathology	Periapical lesions, including periapical radiolucencies, features of inflammation of the periapical tissues, apical periodontitis, and widening of the periodontal ligament space in the apical region.
Periodontal Bone Loss/Perio Widening	Radiological features of periodontal bone loss around the tooth; widening of the periodontal ligament space; features of overload, trauma, or periodontitis; and changes within the furcation. In both the reference standard and the Diagnocat analyses, only moderate or severe lesions were considered; mild bone loss was not counted by either reader.
Endo-iatrogenic	Complications and artifacts related to endodontic treatment, such as underfilling, overfilling, material extruded beyond the apex, a separated instrument, or other iatrogenic abnormalities.
Implant-Pathology	Pathologies related to an implant, in particular, peri-implant bone loss and features of peri-implantitis.
Tooth Position/Development	Abnormalities of tooth position or development, including impaction, ectopic position, rotations, mesial inclination, supernumerary teeth, and retained root fragments.
Fracture	Suspected fracture or crack of a tooth or root or other traumatic damage to tooth structures.
Other Pathology	Other pathological lesions not fitting the above categories, e.g., odontomas, odontogenic lesions, tumors, and other rare or non-specific abnormalities.
Maxillary Sinus Pathology	Maxillary sinus pathologies visible on panoramic radiographs and CBCT, such as mucosal thickening, presence of fluid, cysts, opacifications, and other abnormalities.
Missing Tooth	An indication denoting the absence of a tooth in a given location. Absence of third molars was not treated as a missing tooth.
No Pathology	An indication denoting the absence of radiological features of pathology within a given tooth or assessed structure. This category was treated as a full-fledged diagnostic indication.

**Table 2:** Per-indication analysis for intraoral radiographs.

Category	GS (*n*)	AI (*n*)	TP	FN	FP	Sensitivity	Precision	F1
Caries/Crown Defect	20	22	14	6	8	0.70	0.64	0.67
Apical Pathology	5	6	4	1	2	0.80	0.67	0.73
Periodontal Bone Loss/PDL Widening	3	9	1	2	8	0.33	0.11	0.17
Endo-iatrogenic	7	8	7	0	1	1.00	0.88	0.93
Tooth Position/Development	1	0	0	1	0	0.00	—	0.00
Fracture	1	0	0	1	0	0.00	—	0.00
Other Pathology	4	0	0	4	0	0.00	—	0.00
Missing Tooth	3	4	3	0	1	1.00	0.75	0.86
Total	44	49	29	15	20	0.66	0.59	0.62

GS—reference standard; AI—artificial intelligence software; TP—concordant indications; FN—indications present in the reference standard but not detected by the AI; FP—AI indications not confirmed in the reference standard; F1—harmonic mean of sensitivity and precision. Precision and F1 are not defined where the software recorded no indication in a category and are marked “—”.

**Table 3:** Per-indication analysis for panoramic radiographs.

Category	GS (*n*)	AI (*n*)	TP	FN	FP	Sensitivity	Precision	F1
Caries/Crown Defect	46	52	27	19	25	0.59	0.52	0.55
Apical Pathology	12	12	8	4	4	0.67	0.67	0.67
Periodontal Bone Loss/PDL Widening	36	36	25	11	11	0.69	0.69	0.69
Endo-iatrogenic	19	18	14	5	4	0.74	0.78	0.76
Tooth Position/Development	18	13	11	7	2	0.61	0.85	0.71
Maxillary Sinus Pathology	9	3	3	6	0	0.33	1.00	0.50
Other Pathology	12	3	3	9	0	0.25	1.00	0.40
Missing Tooth	32	34	30	2	4	0.94	0.88	0.91
Total	184	171	121	63	50	0.66	0.71	0.68

GS—reference standard; AI—artificial intelligence software; TP—concordant indications; FN—indications present in the reference standard but not detected by the AI; FP—AI indications not confirmed in the reference standard; F1—harmonic mean of sensitivity and precision.

**Table 4:** Per-indication analysis for CBCT examinations.

Category	GS (*n*)	AI (*n*)	TP	FN	FP	Sensitivity	Precision	F1
Caries/Crown Defect	10	29	10	0	19	1.00	0.34	0.51
Apical Pathology	19	30	16	3	14	0.84	0.53	0.65
Periodontal Bone Loss/PDL Widening	6	20	5	1	15	0.83	0.25	0.38
Endo-iatrogenic	23	23	21	2	2	0.91	0.91	0.91
Tooth Position/Development	14	12	10	4	2	0.71	0.83	0.77
Implant-Pathology	0	1	0	0	1	—	0.00	0.00
Maxillary Sinus Pathology	13	13	11	2	2	0.85	0.85	0.85
Other Pathology	27	40	17	10	23	0.63	0.43	0.51
Missing Tooth	87	64	60	27	4	0.69	0.94	0.79
Total	199	232	150	49	82	0.75	0.65	0.70

GS—reference standard; AI—artificial intelligence software; TP—concordant indications; FN—indications present in the reference standard but not detected by the AI; FP—AI indications not confirmed in the reference standard; F1—harmonic mean of sensitivity and precision. Precision and F1 are not defined where the software recorded no indication in a category and are marked “—”.

**Table 5:** Secondary binary analysis: agreement between the Diagnocat software and the reference standard for intraoral (IO), panoramic (OPG), and CBCT examinations, with one observation per tooth or anatomical site.

Modality	TP	FN	FP	TN	Sensitivity	Specificity	PPV	NPV	Accuracy	F1-Score
IO	27	7	11	16	0.79	0.59	0.71	0.70	0.70	0.75
OPG	117	44	32	236	0.73	0.88	0.79	0.84	0.82	0.75
CBCT	141	36	39	238	0.80	0.86	0.78	0.87	0.83	0.79

TP—locations classified as pathological by both readers; FN—locations pathological in the reference standard and not so classified by the AI; FP—locations free of pathology in the reference standard and classified as pathological by the AI; TN—locations classified as free of pathology by both readers; PPV—positive predictive value; NPV—negative predictive value; F1-score—harmonic mean of sensitivity and precision. Values are point estimates reported descriptively. The counting rules are given in Section 2.5 and the full derivation in Supplementary Material S2.

## Data Availability

The data presented in this study are available on request from the corresponding author.

## Supplementary Materials

The following supporting information can be downloaded at: https://www.mdpi.com/article/10.3390/jcm15207835/s1, Supplementary Material S1, Completed STARD 2015 checklist; Supplementary Material S2, Reconciliation of the diagnostic-indication counts with the binary matrix for each imaging modality.
