# Supplementary material for: Diagnostic Accuracy of Artificial Intelligence Software in Intraoral, Panoramic, and CBCT Imaging: A Pilot Study
Source: J Clin Med. 2026 Oct 11;15(20):7835. doi: 10.3390/jcm15207835 (PMC13705470; doi:10.3390/jcm15207835)
Supplement: Supplementary file 1 [file jcm-15-07835-s001.zip › Supplementary Material S1.pdf]

Supplementary Material S1. STARD 2015 checklist

Diagnostic Accuracy of Artificial-Intelligence Software in Intraoral, Panoramic, and CBCT Imaging: A Pilot Study

Reporting follows the STARD 2015 guidelines (Bossuyt et al., BMJ 2015;351:h5527). Section references correspond to the revised manuscript; page/line numbers to be completed on the final proof.

No.	STARD 2015 item	Reported	Location in manuscript / comment
**TITLE OR ABSTRACT**			
1	Identification as a study of diagnostic accuracy using at least one measure of accuracy (e.g., sensitivity, specificity, predictive values).	Yes	Title; Abstract
2	Structured summary of study design, methods, results, and conclusions.	Yes	Abstract
**INTRODUCTION**			
3	Scientific and clinical background, including the intended use and clinical role of the index test.	Yes	Introduction
4	Study objectives and hypotheses.	Yes	Introduction (final paragraph — objective and hypothesis)
**METHODS**			
5	Whether data collection was planned before the index test and reference standard were performed (prospective) or after (retrospective).	Yes	Section 2.1 — retrospective
6	Eligibility criteria.	Yes	Section 2.1 — anonymized, diagnostic-quality examinations; tooth-germ findings excluded
7	On what basis potentially eligible participants were identified (e.g., symptoms, previous tests, registry).	Yes	Section 2.1 — selected independently of the presence/absence of pathology
8	Where and when potentially eligible participants were identified (setting, location, dates).	Yes	Section 2.1 — two centers (Bytom; Goczałkowice-Zdrój); analysis December 2025
9	Whether participants formed a consecutive, random, or convenience series.	Yes	Section 2.1 — convenience (non-systematic) series
10a	Index test, in sufficient detail to allow replication.	Yes	Section 2.3 — Diagnocat, December 2025 version, default settings
10b	Reference standard, in sufficient detail to allow replication.	Yes	Section 2.2 — whole-image assessment by an expert with >30 years' experience
11	Rationale for choosing the reference standard (if alternatives exist).	Yes	Section 2.2 — expert radiological reading adopted as reference in the absence of histopathology
12a	Definition of and rationale for index-test positivity cut-offs/result categories, distinguishing prespecified from exploratory.	Yes	Section 2.3 — prespecified 50% (default 'more-likely-than-not') threshold
12b	Definition of and rationale for reference-standard positivity cut-offs/result categories, distinguishing prespecified from exploratory.	Yes	Section 2.4; Table 1 — prespecified diagnostic-category definitions
13a	Whether clinical information and reference-standard results were available to the performers/readers of the index test.	Yes	Sections 2.1, 2.3 — automated analysis without clinical data or reference-standard access
13b	Whether clinical information and index-test results were available to the assessors of the reference standard.	Yes	Section 2.2 — expert blinded to AI output, source center, and patient identity
14	Methods for estimating or comparing measures of diagnostic accuracy.	Yes	Section 2.6 — sensitivity/specificity/PPV/NPV/accuracy/F1; Pearson χ^2; Wilson 95% CI; cross-modality χ^2
15	How indeterminate index-test or reference-standard results were handled.	Yes	Section 2.4 — no examination produced an indeterminate result for either test; classifications were binary (Section 2.3)
16	How missing data on the index test and reference standard were handled.	Yes	Section 2.5, rule (6) and Figure 1 — the software produced no entry at 23 of the locations charted by the expert; 22 were counted as false negatives and 1 as a true negative, the conservative choice. Listed in S2, section E, with a sensitivity analysis excluding them.

			No missing reference-standard results
17	Any analyses of variability in diagnostic accuracy, distinguishing prespecified from exploratory.	Yes	Sections 2.6, 3.5 — cross-modality comparison reported as exploratory
18	Intended sample size and how it was determined.	Yes	Section 2.1 — feasibility sample of 15/modality; no formal power calculation (stated)
**RESULTS**			
19	Flow of participants, using a diagram.	Yes	Figure 1 — STARD flow diagram (screened / excluded / included)
20	Baseline demographic and clinical characteristics of participants.	Partly	Section 2.1 — n = 45 (one examination per patient); age and sex unavailable owing to anonymization (Limitations)
21a	Distribution of severity of disease in those with the target condition.	Partly	Tables 2–4 — category distribution reported; severity not separately graded (Limitations)
21b	Distribution of alternative diagnoses in those without the target condition.	Yes	Tables 2–5 — 'No pathology' and per-category distribution
22	Time interval and any clinical interventions between index test and reference standard.	N/A	Both the index test and the reference standard assessed the same images; no time interval or intervention (Section 2.1)
23	Cross-tabulation of the index-test results by the results of the reference standard.	Yes	Tables 2–5 — 2×2 'pathology vs. no pathology' matrices and per-category TP/FN/FP
24	Estimates of diagnostic accuracy and their precision (e.g., 95% CI).	Yes	Section 2.6; Tables 2–5 — point estimates with Wilson 95% CIs
25	Any adverse events from performing the index test or the reference standard.	N/A	Retrospective analysis of anonymized images; no patient contact and no adverse events
**DISCUSSION**			
26	Study limitations, including sources of potential bias, statistical uncertainty, and generalizability.	Yes	Discussion (Limitations) — single-reader standard, convenience sampling, clustering, single AI/threshold
27	Implications for practice, including the intended use and clinical role of the index test.	Yes	Discussion; Conclusions
**OTHER INFORMATION**			
28	Registration number and name of registry.	N/A	The study was not prospectively registered
29	Where the full study protocol can be accessed.	Yes	Available from the corresponding author on request
30	Sources of funding and other support; role of funders.	Yes	Funding statement — no external funding

References

Bossuyt, P.M.; Reitsma, J.B.; E Bruns, D.; A Gatsonis, C.; Glasziou, P.P.; Irwig, L.; Lijmer, J.G.; Moher, D.; Rennie, D.; de Vet, H.C.W.; et al. STARD 2015: an updated list of essential items for reporting diagnostic accuracy studies. BMJ 2015, 351, h5527, https://doi.org/10.1136/bmj.h5527.
