# Supplementary material for: Diagnostic Accuracy of Artificial Intelligence Software in Intraoral, Panoramic, and CBCT Imaging: A Pilot Study
Source: J Clin Med. 2026 Oct 11;15(20):7835. doi: 10.3390/jcm15207835 (PMC13705470; doi:10.3390/jcm15207835)
Supplement: Supplementary file 1 [file jcm-15-07835-s001.zip › Supplementary Material S2.pdf]

# Supplementary Material S2

Derivation of the reported counts, reconciliation of the two analyses, and record of data corrections

This document sets out how every reported count was obtained, so that the analysis can be reproduced from the source records. The counting and classification rules are stated in Section 2.5 of the manuscript and are applied here without exception. Examinations are identified by modality and by an anonymized code (F = female, M = male).

## A. Analysis set

| | Intraoral | Panoramic | CBCT | Total |
|---|---|---|---|---|
| Examinations | 15 | 15 | 15 | 45 |
| Diagnostic indications, reference standard | 71 | 452 | 476 | 999 |
| Diagnostic indications, software | 69 | 444 | 504 | 1017 |
| Locations charted by the reference standard (unit of the binary analysis) | 61 | 429 | 454 | 944 |
| of which with no software entry | 3 | 10 | 10 | 23 |
| Locations reported by the software only (excluded) | 1 | 2 | 8 | 11 |

## B. The two analyses side by side

The primary analysis counts diagnostic indications; the secondary binary analysis counts locations. The totals differ by construction and are not expected to coincide.

| | Intraoral | Panoramic | CBCT |
|---|---|---|---|
| Primary: pathological indications, reference standard | 44 | 184 | 199 |
| Primary: true positives | 29 | 121 | 150 |
| Primary: false negatives | 15 | 63 | 49 |
| Primary: false positives | 20 | 50 | 82 |
| Secondary: true positives | 27 | 117 | 141 |
| Secondary: false negatives | 7 | 44 | 36 |
| Secondary: false positives | 11 | 32 | 39 |
| Secondary: true negatives | 16 | 236 | 238 |
| Secondary: total locations | 61 | 429 | 454 |

## C. Worked example

Tooth 48 of panoramic examination F1. Reference standard: caries/crown defect and periodontal bone loss. Software: caries/crown defect and apical pathology. Primary analysis: one true positive, one false negative and one false positive, that is three indications from one tooth. Secondary analysis: both readers classify the location as pathological, so it contributes one true positive.

Tooth 22 of panoramic examination F3. Reference standard: apical pathology. Software: caries/crown defect. Primary analysis: one false negative and one false positive. Secondary analysis: one true positive, because both readers classify the location as pathological irrespective of category.

A tooth for which both readers recorded only "No pathology" contributes one concordant negative indication to the primary analysis and one true negative to the secondary analysis.

## D. Composition of the false negatives in the secondary analysis

False negatives arise in two distinct ways, which are reported separately because they have different causes.

| | Intraoral | Panoramic | CBCT | Total |
|---|---|---|---|---|
| Software produced no entry for the location | 3 | 10 | 9 | 22 |
| Software charted the location but classified it as free of pathology | 4 | 34 | 27 | 65 |
| Total false negatives | 7 | 44 | 36 | 87 |

## E. Locations charted by the reference standard with no software entry

The software does not report on every anatomical location, so an absent entry does not mean that the location was assessed and found normal. Twenty-two of these locations were positive in the reference standard and were counted as false negatives, which is the conservative choice; one was negative and was counted as a true negative.

| Modality | Examination | Location | Reference standard | Counted as |
|---|---|---|---|---|
| Intraoral | F3 | tooth 45 | Caries / crown defect | False negative |
| Intraoral | M4 | examination level | Other pathology | False negative |
| Intraoral | M5 | examination level | Other pathology | False negative |
| Panoramic | F2 | examination level | Other pathology | False negative |
| Panoramic | F2 | tooth 47 | Missing tooth | False negative |
| Panoramic | F4 | tooth 14 | Missing tooth | False negative |
| Panoramic | M2 | right maxillary sinus | Maxillary sinus pathology | False negative |
| Panoramic | M3 | tooth 43 | Tooth position / development | False negative |
| Panoramic | M3 | right maxillary sinus | Maxillary sinus pathology | False negative |
| Panoramic | M6 | right maxillary sinus | Maxillary sinus pathology | False negative |
| Panoramic | M8 | right maxillary sinus | Maxillary sinus pathology | False negative |
| Panoramic | M9 | left maxillary sinus | Maxillary sinus pathology | False negative |
| Panoramic | M9 | right maxillary sinus | Maxillary sinus pathology | False negative |
| CBCT | F1 | right parapharyngeal space | Other pathology | False negative |
| CBCT | F1 | left parapharyngeal space | Other pathology | False negative |
| CBCT | F2 | tooth 38 | Tooth position / development | False negative |
| CBCT | F2 | tooth 48 | Tooth position / development | False negative |
| CBCT | F3 | tooth 48 | No pathology | True negative |
| CBCT | F3 | right maxillary sinus | Maxillary sinus pathology | False negative |
| CBCT | F8 | right maxillary sinus | Maxillary sinus pathology | False negative |
| CBCT | M3 | mesiodens | Tooth position / development | False negative |
| CBCT | M3 | incisive canal cyst | Other pathology | False negative |
| CBCT | M6 | mesiodens | Tooth position / development | False negative |

The maxillary sinus accounts for six of the ten panoramic locations and two of the ten CBCT locations. The exclusion of third molars applies only to the Missing tooth category; indications of position or development recorded on teeth 38 and 48 are therefore analysed in the same way as indications on other teeth.

In the terms of STARD 2015 these 23 locations are missing index-test results at the level of the individual location, and they are reported as such in Figure 1 of the manuscript. They are not missing at the level of the examination: the software returned a complete report for all 45 examinations.

## F. Locations reported by the software only

These locations were excluded from the binary analysis because the reference standard assigns them no status.

| Modality | Examination | Location | Software indication |
|---|---|---|---|
| Intraoral | M1 | tooth 35 | Missing tooth |
| Panoramic | F1 | tooth 17 | Caries / crown defect |
| Panoramic | M2 | tooth 18 | Caries / crown defect |
| CBCT | F1 | tooth 17 | Periodontal bone loss |
| CBCT | F1 | left maxillary sinus | Maxillary sinus pathology |
| CBCT | F5 | tooth 48 | Tooth position / development |
| CBCT | F8 | tooth 12 | Other pathology |
| CBCT | F8 | tooth 23 | Apical pathology |
| CBCT | F8 | tooth 24 | Other pathology |
| CBCT | F8 | tooth 33 | Caries / crown defect; periodontal bone loss |
| CBCT | M4 | left maxillary sinus | Maxillary sinus pathology |

## G. Correction of the panoramic reference standard

Two locations in the panoramic reference standard carried both a pathological indication and a "No pathology" label. Who resolved them, how and when is stated here in full, because the resolution was made after the software output was known.

The two conflicts were resolved by the first author (W.C.), against the expert's original source records for those two examinations. The radiographs themselves were not re-read for this purpose, and the expert who established the reference standard took no part in this step and remained blinded to the software output throughout. The resolution was carried out during the revision of the manuscript, at a stage when the software output was already available to the first author.

| Record | Action | Effect |
|---|---|---|
| F1, tooth 12, caries/crown defect (reference standard) | Removed; the source record for this examination does not support a carious lesion at this tooth | Reference indications 454 to 452 |
| F4, tooth 34, "No pathology" (reference standard) | Removed; the source record confirms the endo-iatrogenic indication | The location remains positive for the reference standard |

| Caries / Crown defect category, panoramic | Before | After |
|---|---|---|
| Reference standard indications | 47 | 46 |
| True positives | 28 | 27 |

The resolution did not move the reference standard towards the software. At tooth 12 of examination F1 the software had recorded a carious lesion, and the location was nevertheless resolved as free of pathology; that software indication is therefore counted as a false positive, so the decision counts against the software rather than in its favour.

The software output was left exactly as produced. Three locations in the software output carried both a pathological indication and a "No pathology" label: tooth 12 of panoramic examination F1, tooth 46 of panoramic examination M3 and tooth 18 of CBCT examination M1. Under the rule stated in Section 2.5 all three were classified as positive for the software, without exception.

## H. Correction of the CBCT reference standard

During the verification of the source records carried out in response to the reviewers, the edentulous areas described in the reference report of one CBCT examination were found not to have been transcribed tooth by tooth into the structured dataset. The report describes a free-end edentulous area in the upper right quadrant, an edentulous area in the upper left quadrant with post-extraction sockets, an interdental gap in the lower left quadrant and an interdental gap at tooth 46. The corresponding eleven missing-tooth indications were completed by the first author (W.C.) from that written report together with the teeth the expert had charted as present. Third molars were not included, in accordance with the protocol.

The circumstances of this correction are stated in full because it increases the agreement between the two readers. It was made during the revision of the manuscript, at a stage when the software output was already available to the first author. The software output was not used to derive the eleven FDI numbers, but those numbers coincide exactly with the eleven missing teeth reported by the software for this examination. Both the pre-correction and the post-correction values are given below, and the pre-correction values are also reported in Section 3.5 of the manuscript as a sensitivity analysis, so that the effect of the correction is visible to the reader.

| | **Before** | **After** |
|---|---|---|
| CBCT reference-standard indications | 465 | 476 |
| Missing tooth category: reference indications | 76 | 87 |
| Missing tooth category: true positives | 49 | 60 |
| Missing tooth category: false negatives | 27 | 27 |
| Missing tooth category: false positives | 15 | 4 |
| Binary analysis: true positives | 130 | 141 |
| Binary analysis: total locations | 443 | 454 |
| Binary analysis: sensitivity | 0.78 | 0.80 |
| Binary analysis: specificity | 0.86 | 0.86 |
| Binary analysis: accuracy | 0.83 | 0.83 |
| Binary analysis: F1-score | 0.78 | 0.79 |
| Locations reported by the software only | 19 | 8 |

All other examinations were checked for edentulous areas described in the reference report but not transcribed tooth by tooth, and none was found. The table below gives the missing-tooth counts of both readers for each of the 15 CBCT examinations, so that this check can be verified directly.

| **Examination** | **Reference standard** | **Software** | **TP** | **FN** | **FP** |
|---|---|---|---|---|---|
| F1 | 6 | 7 | 6 | 0 | 1 |
| F2 | 0 | 0 | 0 | 0 | 0 |
| F3 | 9 | 0 | 0 | 9 | 0 |
| F4 | 13 | 2 | 2 | 11 | 0 |
| F5 | 2 | 2 | 1 | 1 | 1 |
| F6 | 23 | 22 | 22 | 1 | 0 |
| F7 | 3 | 4 | 2 | 1 | 2 |
| F8 | 11 | 11 | 11 | 0 | 0 |
| M1 | 2 | 2 | 2 | 0 | 0 |
| M2 | 0 | 0 | 0 | 0 | 0 |
| M3 | 6 | 4 | 4 | 2 | 0 |
| M4 | 0 | 0 | 0 | 0 | 0 |
| M5 | 3 | 3 | 3 | 0 | 0 |
| M6 | 1 | 0 | 0 | 1 | 0 |
| M7 | 8 | 7 | 7 | 1 | 0 |
| Total | 87 | 64 | 60 | 27 | 4 |

The totals of this table are the Missing tooth row of Table 4 of the manuscript. The difference between the column totals, 87 against 64, is concentrated in three examinations: F3, where the expert recorded nine missing teeth and the software none; F4, where the expert recorded thirteen and the software two; and M3, where the difference is two. In all three the reference standard holds more missing teeth than the software, so these are failures of the software to report edentulous areas, counted as false negatives, and not untranscribed records on the reference side. The problem corrected in examination F8 ran in the opposite direction, the reference standard holding fewer entries than the report described, and F8 was the only examination in which it occurred. In the remaining twelve examinations the two readers differ by at most one.

## I. Sensitivity analyses

Missing tooth category excluded:

| Modality | TP | FN | FP | TN | N | Sensitivity | Specificity | Accuracy | F1 |
|---|---|---|---|---|---|---|---|---|---|
| Intraoral | 24 | 7 | 11 | 16 | 58 | 0.77 | 0.59 | 0.69 | 0.73 |
| Panoramic | 86 | 45 | 29 | 239 | 399 | 0.66 | 0.89 | 0.81 | 0.70 |
| CBCT | 77 | 15 | 35 | 242 | 369 | 0.84 | 0.87 | 0.86 | 0.75 |

All locations with no software entry excluded:

| Modality | TP | FN | FP | TN | N | Sensitivity | Specificity | Accuracy | F1 |
|---|---|---|---|---|---|---|---|---|---|
| Intraoral | 27 | 4 | 11 | 16 | 58 | 0.87 | 0.59 | 0.74 | 0.78 |
| Panoramic | 117 | 34 | 32 | 236 | 419 | 0.77 | 0.88 | 0.84 | 0.78 |
| CBCT | 141 | 27 | 39 | 237 | 444 | 0.84 | 0.86 | 0.85 | 0.81 |

Sensitivity is higher in this second analysis because the locations on which the software does not report are removed from the denominator. The values reported in the manuscript are the more conservative ones, in which these locations are retained and counted against the software.

## J. Note on measures of precision

All values are point estimates reported descriptively. No confidence intervals and no hypothesis tests are given, because diagnostic indications and locations are clustered within examinations and patients and, with 15 examinations per modality, methods assuming independent observations would produce intervals that are too narrow.
